# Development of Protein- and Peptide-Based HIV Entry Inhibitors Targeting gp120 or gp41

**DOI:** 10.3390/v11080705

**Published:** 2019-08-01

**Authors:** Jing Pu, Qian Wang, Wei Xu, Lu Lu, Shibo Jiang

**Affiliations:** 1 Shanghai Public Health Clinical Center and School of Basic Medical Sciences, Key Laboratory of Medical Molecular Virology (MOE/NHC/CAMS), Fudan University, Shanghai 200032, China; 2 Lindsley, F. Kimball Research Institute, New York Blood Center, New York, NY 10065, USA

**Keywords:** HIV-1, gp120, gp41, entry inhibitor, peptide, antibody, recombinant protein, antiretroviral drugs

## Abstract

Application of highly active antiretroviral drugs (ARDs) effectively reduces morbidity and mortality in HIV-infected individuals. However, the emergence of multiple drug-resistant strains has led to the increased failure of ARDs, thus calling for the development of anti-HIV drugs with targets or mechanisms of action different from those of the current ARDs. The first peptide-based HIV entry inhibitor, enfuvirtide, was approved by the U.S. FDA in 2003 for treatment of HIV/AIDS patients who have failed to respond to the current ARDs, which has stimulated the development of several series of protein- and peptide-based HIV entry inhibitors in preclinical and clinical studies. In this review, we highlighted the properties and mechanisms of action for those promising protein- and peptide-based HIV entry inhibitors targeting the HIV-1 gp120 or gp41 and discussed their advantages and disadvantages, compared with the current ARDs.

## 1. Introduction

According to UNAIDS, approximately 36.9 million people worldwide were living with human immunodeficiency virus (HIV) in 2017 (https://www.unaids.org). The introduction of highly active antiretroviral drugs (ARDs) mainly includes nucleoside/nonnucleoside reverse transcriptase inhibitors (NRTIs/NNRTIs), protease inhibitors (PIs), and integrase inhibitors (INIs). However, the long-term use of ARDs has caused the emergence of multi-drug resistant HIV strains, resulting in more and more treatment failure [1,2]. In 2003, the first peptide-based HIV entry inhibitor, enfuvirtide, was approved by the U.S. FDA for clinical use to treat HIV/AIDS patients who failed to respond to the current ARDs. Most ARDs must enter the host cells to target the enzymes required for HIV replication. Instead, HIV entry inhibitors do not enter the host cell, but rather, act outside the cells and block HIV entry into the target cells by interacting with the envelope glycoproteins (Env) on the surface of the virion. So far, a series of protein- and peptide-based HIV entry inhibitors have been developed in preclinical and clinical studies.

**Figure 1:**
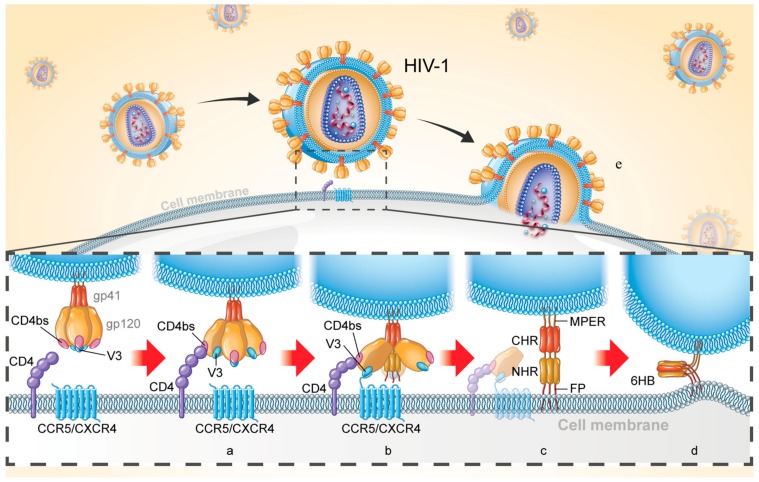
Schematic diagram of HIV-1 entry into target cell. (**a**) Binding of gp120 to CD4 receptor; (**b**) Binding of gp120 to the coreceptor CCR5 / CXCR4; (**c**) Formation of prehairpin intermediate (PFI) and connection of viral membrane and cell membrane; (**d**) Formation of six-helix bundle (6HB); (**e**) HIV-1 releases its genome into target cells.

HIV-1 Env (approximately 160 kD, also known as gp160) is comprised of two noncovalently bound subunits (surface subunit gp120 and transmembrane subunit gp41) upon maturity and plays key roles in viral entry. Such entry is initiated by binding of gp120 to the CD4 receptor (Figure 1a), inducing viral recognition, proximity to the cell, exposure of the coreceptor binding sites (CoRbs), CCR5 or CXCR4 (Figure 1b). A conformational change of the gp120-gp41 complex results in the release of gp41 subunit and triggers fusion of the viral-cell membrane. Specifically, fusion peptide (FP) inserts into the target cell membrane and forms an extended prehairpin intermediate (PHI) conformation and links the virus to the cell membrane (Figure 1c). PHI undergoes a further conformational change in which three N-terminal heptad repeats (NHRs) form the inner core and then three C-terminal heptad repeats (CHRs) encapsulate the NHR trimer in an anti-parallel manner to form a six-helix bundle (6HB) (Figure 1d), thereby forming a fusion pore and causing release of the HIV-1 genome for target cell entry (Figure 1e). 

A variety of substances work cooperatively and synergistically during viral-cell membrane fusion and create complex interactive networks involving a variety of protein-protein interactions, such as CD4-gp120 [3,4,5], gp120-CCR5 / CXCR4 [6], gp120-gp41 [7,8], gp41 NHR-CHR [9], and interaction between the intracellular and extracellular regions of gp41 [10]. Entry inhibitors target these proteins, their interfaces, or other sites to block viral invasion and can be divided into three major subclasses: adhesion inhibitors, targeting CD4 or gp120 to block CD4-gp120 interaction; coreceptor inhibitors, targeting CCR5 or CXCR4 to inhibit the binding of gp120 to the coreceptor; and fusion inhibitors, targeting gp41 to interfere with its conformational change required for viral fusion and entry. In addition, inhibitors targeting both gp120 and gp41, which may have stronger inhibitory activity and higher genetic barrier, are also at the forefront of current research. In this review, we will discuss the characteristics of protein- and peptide-based inhibitors that specifically target HIV Env and look ahead to their development.

## 2. Protein-and Peptide-Based HIV Entry Inhibitors Targeting gp120

**Figure 2:**
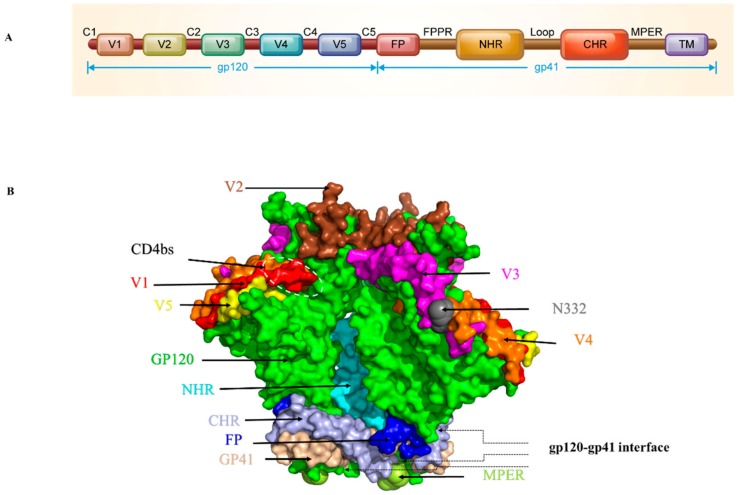
Ectodomain structure of HIV-1 Env trimer. (**A**) Schematic diagram of HIV-1 gp120 and gp41. (**B**) Side view of the Env trimer. Gp120 is colored in green with the V1, V2, V3, V4, and V5 regions colored in red, brown, purple, orange and yellow, respectively. CD4bs is marked with a white dotted frame. Gp41 is colored in light pink with the fusion peptide (FP), N-terminal heptad repeats (NHR), C-terminal heptad repeats (CHR) and membrane-proximal external region (MPER) regions colored in blue, cycan, bright gray and cycan-blue, respectively. The images were generated with the software PyMOL according to the PDB ID: 5VJ6.

The HIV-1 Env surface subunit gp120 contains 5 conserved constant regions (C1-C5) and 5 variable regions (V1-V5) and plays an important role in initiating and controlling HIV-1 infection [6,11,12] (Figure 2A). The N332 glycoprotein, CD4-binding site (CD4bs), and V1-2 loops are conserved sites targeted by many antibodies (Figure 2B). Recently, the structure of the gp120 V3 loop interacting with the chemokine binding pocket in CCR5 and the interaction of the CD4-induced bridging sheet of gp120 with the N-terminus of CCR5 have been resolved, revealing key coreceptor binding residues in gp120 [6], providing detailed structural information for gp120-coreceptor interaction. This key structural information provides the basis for analyzing the interaction between inhibitors and viruses, as well as helping to further the design of optimized inhibitors.

### 2.1. Antibodies or Recombinant Proteins Targeting gp120

#### 2.1.1. Antibodies or Recombinant Proteins Targeting gp120 CD4bs

##### The gp120 CD4bs-Targeting Antibodies

VRC01, VRC02, and VRC03 are broad-spectrum neutralizing antibodies isolated from B cells of HIV-1-infected individuals, partially mimicking the interaction of CD4 receptor with gp120 [13]. Studies have shown that VRC01 interacts primarily with three regions of gp120, including loop D, CD4bs, and V5, causing a conformational change in gp120 such that its binding to CD4 receptor is blocked. In terms of neutralizing activity, VRC01 is similar to that of VRC02 and greater than that of VRC03 [14]. Unfortunately, the cell-cell fusion inhibitory activity of VRC01 is significantly reduced [15], and VRC01-resistant strains appeared [16,17,18]. NIH45-46 are more potent VRC01 mutants screened from the HIV-1 antibody library using single-cell cloning technology [19]. Both are from the same patient and currently undergoing clinical trials. NIH45-46^G54W^ is a structure-based NIH45-46 mutant that enhances interaction with the gp120 bridging sheet. Compared with NIH45-46 and VRC01, NIH45-46^G54W^ has a wider neutralization profile and a 10-fold or 20-fold increase in neutralizing activity [20]. 45-46 m2 and 45-46 m7 are NIH45-46^G54W^ mutants based on the NIH45-46-gp120 complex structure and the NIH45-46^G54W^ resistant strain sequence analysis design. The activity of 45-46 m2 was similar to that of NIH45-46^G54W^, and it was greater than that of 45-46 m7. However, the 45-46 m2-neutralizing NIH45-46 resistant strain was more effective than NIH45-46^G54W^ [21]. Combination therapy of 45-46 m2 and 45-46 m7 in mice infected with HIV-1 reduced viral escape. N6 is a bNAb isolated from an HIV-infected patient, and it targets the gp120 CD4bs. It has more effective neutralizing activity than VRC01, and inhibits the VRC01-resistant strain. This could be attributed to the dependence of N6 on the conserved region of gp120 and avoidance of the interaction of its light chain with the gp120 V5 region, which is considered to be a major factor in the resistance of the virus to VRC01-class antibodies [22]. In a non-human primate model, mutants of N6 show significant antiviral effects [23]. Currently, N6 is undergoing clinical trials to assess whether it can safely inhibit HIV-1 infection in humans. 3BNC117 is an HIV-1 patient-derived bNAb that targets the gp120 CD4bs [19]. The results of non-human primates showed that 3BNC117 or 3BNC117 combined with PGT121 significantly reduced the viral load of SHIV [24,25] and that it had long-term protective effects against repeated low-dose SHIV challenges [26]. The first phase of clinical trials showed that 3BNC117 has good pharmacokinetic properties and is effective in inhibiting viremia in HIV-1-infected patients for up to 28 days, although some patients developed 3BNC117-resistant strains in a short period of time [27]. It is worth noting that passive immunization of 3BNC117 accelerates the clearance of infected cells [28]. Phase IIa clinical trial data show that 3BNC117 can effectively inhibit viral rebound in patients with treatment interruption [29]. In addition, cellular immunity and humoral immunity to HIV-1 were found in patients treated with 3BNC117 [30,31]. PGV04 (also known as VRC-PG04) is a gp120 CD4bs-targeting bNAb isolated from an elite controller infected with an HIV-1 clade A1/D recombinant [32]. It exhibits broad-spectrum and neutralizing activity similar to that of VRC01 and 3BNC117, but the latter two were isolated from patients infected with a single subtype of HIV-1; therefore, the induction of bNAb targeting CD4bs is not dependent on viral subtype because these CD4bs-specific bNAbs have different CD4bs recognition patterns and different ways to access the CD4bs [33].

F105, an IgG1 kappa antibody targeting a discontinuous epitope on gp120, was identified in the early 1990s. Subsequently, the neutralization activity was evaluated [34]. The co-crystal structure shows that its conformation for gp120 recognition is poorly compatible with virus spikes, resulting in low neutralizing activity [35]. Although it has been more than a decade since the clinical trials were carried out, no progress has occurred for a long time.

Human mAb b12, which targets CD4bs of the gp120, could neutralize diverse HIV-1 primary isolates and fully protects hu-PBL-SCID mice from HIV-1 infection in vivo [36,37]. Interestingly, b12 Fab exerts inhibitory activity upon entry of the virus into cells. Therefore, b12 is thought to have a dual role, acting before and after infection [38]. Subsequently, the structure of b12 and Env was extensively studied, providing a basis for epitope studies of other bNAbs [39,40,41]. 

##### The gp120 CD4bs-Targeting Proteins

The binding of gp120 to CD4 plays a key role in the first step of viral entry. Soluble CD4 (sCD4), which contained four extracellular immunoglobulin homology domains (T4), can compete with the CD4 receptor for ligand and was the earliest candidate to be considered for development into potential anti-HIV drugs [42]. It was found that the first two extracellular domains (D1D2) could also simulate CD4 receptor binding to gp120 [43]. Then a series of antiviral tests were carried out in cells, animal models and clinical trials [44,45,46]. Results showed that sCD4 had high anti-HIV-1 activity in vitro and in vivo [47,48]. However, the half-life of sCD4 was relatively short. In some patients, the virus rebounded quickly and showed no evident treatment effects [44,45,46]. In addition, sCD4, at low concentrations, could enhance the infection of some clinical strains, possibly because the binding of sCD4 to HIV-1 gp120 results in the exposure of CoRbs, making it easy for the virus to infect neighboring cells [49,50]. These drawbacks greatly reduced the clinical applications of sCD4. In order to block the interaction between gp120 and the CD4 receptor in a more effective way, PRO-542, which contained the binding domain of CD4 (D1D2) and an IgG2 scaffold without the VH and VL domains, was designed [51,52]. As expected, PRO-542 interrupted CD4 binding with gp120 by mimicking the CD4 receptor and enhancing peptide valence and flexibility. Furthermore, it possessed broad neutralizing activity against primary HIV-1 isolates in vitro, and could decrease the viral load in murine models [53,54]. Compared to sCD4, PRO-542 bound with more avidity to virions and had a long half-life of 3-4 days in vivo. A Phase II single-dose study showed that PRO 542 caused an 80% response rate and that viral load was significantly reduced for several weeks post-treatment. However, intravenous administration and drug cost may be obstacles to clinical use [51,55]. Some smaller forms of sCD4 that only contain the D1 domain were designed based on the crystal structure of CD4 and gp120. However, they were very unstable in neutral environment and had lower affinity with gp120 than D1D2 [56]. Recently, using the power of a large phage display library and error-prone PCR technology, Dr. Dimitrov and colleagues identified two stable D1 mutants (mD1.1 and mD1.2), which could be expressed abundantly in *E. coli*, with high solubility and stability in neutral environment. In vitro antiviral experiments displayed that mD1.1 and mD1.2 could inhibit multiple HIV-1 strains at low nM level. They not only exhibited lower binding with human blood cell lines, but also resisted trypsin digestion and human serum degradation [57]. This group also identified an mD1.2 mutant with A55V mutation, mD1.22, from the phage display library, which exhibited better lytic expression, thermostability, specificity and neutralizing activity than mD1.2 [58]. Subsequently, they generated several bifunctional multivalent proteins (2Dm2m, 4Dm2m and 6Dm2m) by linking mD1.22 with a gp120 CoRbs-targeting neutralizing antibody domain (m36.4) and human IgG1 Fc [58]. Because these fusion proteins target both the CD4bs and CoRbs, they exhibited broadly cross-reactive and exceptionally potent neutralizing activity, about 10-, 50-, and 200-fold more potent than the bNAb VRC01, T20, and CD4-Ig, respectively. Furthermore, they have shown higher stability and specificity and a lower aggregation propensity than CD4-Ig. Later, they constructed a 4Dm2m mutant with prolonged half-life in mouse serum [59]. Most recently, we have tested the anti-HIV-1 activity of 4Dm2m in combination with several currently used ARDs, including Zidovudine (NRTI), Efavirenz (NNRTI), or Saquinavir (protease inhibitor), and with the gp41-targeting peptides, including T20, T2635, or SFT. We found that all these combinations exhibited synergistic anti-HIV-1 activity against infection by laboratory-adapted and primary HIV-1 strains, including those resistant to NRTIs and peptide-based HIV-1 entry inhibitors [60]. Application of these combinations are expected to reduce the dose of 4Dm2m and the ARDs tested, thereby reducing treatment costs and cytotoxicity.

##### The gp120 CD4bs-Targeting Peptides

Based on the β-hairpin region of a short scorpion toxin, scyllatoxin, and CDR2-like loop of CD4, CD4M9, a 28 amino acid peptide mimicking CD4 was synthesized [61]. The Phe-43 pocket of gp120 is a conserved and hydrophobic pocket, which binds to the CD4 receptor. CD4M9 can bind to the conserved CD4-binding pocket and thus interfere with CD4-gp120 interactions. With five additional mutations, CD4M9 was shown to bind the Phe-43 pocket of gp120 with nM affinity, effectively inhibiting infection of HIV-1 laboratory-adapted strains [61,62,63]. The characteristics of CD4M9 and its binding gp120 have been studied. Like sCD4, CD4M9 can induce a conformational change of gp120 after its binding to gp120, exposing the binding epitopes of antibody 17b and FabX5, both of which are CD4-induced (CD4i) epitopes, which overlap the CoRbs. Therefore, binding of CD4M9 to gp120 could block binding of gp120 to coreceptor [64]. To optimize interaction with gp120, a 27 amino acid CD4 mimetic peptide, CD4M33, was designed with a CD4-like affinity to HIV-1 Env. It inhibits HIV-1 activity more efficiently than sCD4 and is effective against sCD4-resistant strains [65]. CD4M47, a derivative of CD4M33, has a neutralizing profile similar to that of CD4M33, but without significant improvement in activity [66].

M48 is a CD4 mimetic that targets the gp120 Phe-43 pocket [66], and its derivatives include M48U1-M48U14 [67]. M48U1, composed of 27 amino acids, has been extensively studied. It inhibits the HIV-1 pseudovirus at the pM or nM level [68] and can induce gp120 shedding at high concentrations, resulting in a reduction in new viral particles released by infected cells [69]. In addition, M48U1 is resistant to rhesus macaque infection SHIV [70] and is expected to act as a microbicide to prevent sexual transmission of HIV-1.

Using phage-displayed 12-mer peptide libraries, a novel peptide, G1, which inhibits the interaction between HIV-1 gp120 and CD4 was found. The IC50 for inhibition of the interaction between gp120 and CD4 is about 50 µM. NMR structural analysis showed G1 to be capable of forming a tight ring structure upon binding to gp120. The follow-up results showed that six amino acids (PSFDLQ called G1-6) in the middle of G1 play a crucial role in its activity. This hexametric linear peptide exhibited about 10-fold lower IC50 of 6 µM than G1. Furthermore, adding cysteines on G1-6 at both ends let it form a ring, and the G1-c peptide (CQPSFDLQC) showed an even lower IC50 of about 1 µM [71]. However, in the current literature, we could find no related studies reporting on the inhibitory activity of these peptides on cell-cell fusion, as well as a variety of HIV-1 strains, which, therefore, begs for further assessment.

Using random phage libraries of 12-mer, 7-mer, and cyclin 9-mer peptides, Ferrer and Harrison screened a 12-mer peptide, 12p1, that interacted with gp120 and inhibited the binding of gp120 from three different HIV strains to four-domain soluble CD4 [72]. Interestingly, it could also inhibit the interaction of gp120 and mAb 17b. Analysis of the 12p1 binding site suggested that the inhibitory activity of 12p1 to mutant viruses was reduced significantly by the mutation of Arg476, Lys97 or Glu102 of gp120. These gp120 residues make no contact CD4, but all are located near the CD4bs. These results suggested that the binding mechanisms of CD4 and 12p1 are different. It is possible that 12p1 interacts with and stabilizes an unliganded gp120, rather than the activated one by CD4, resulting in the inability of gp120 to interact with its receptors [73]. Subsequently, the interaction between 12p1 and gp120 was studied by STD NMR, and after models of 12p1 binding to gp120 in different states were proposed, the subtle difference was revealed [74]. Its derivatives showed inhibitory activity at the nM level in viral infection experiments (IC50=156 nM for R5 Bal strain) [75]. However, compared to sCD4, further optimization still needs to be done. In addition, a common drawback among CD4M9, G1 and 12p1 is the human body. That is, modified exogenous proteins are likely to induce specific antibodies which would have a negative impact on the inhibitory activity, or half-life, limiting their clinical use as therapeutic agents. 

#### 2.1.2. Antibodies, Recombinant Proteins and Peptides Targeting gp120 Coreceptor-Binding Site (CoRbs)

##### The gp120 CoRbs-Targeting Antibodies

Some HIV-neutralizing antibodies can recognize the epitope in gp120 induced by CD4 binding. This CD4i epitope generally overlaps with CoRbs on gp120 [76]. Therefore, most of these antibodies inhibit the binding of gp120 to the coreceptor. M36 is a single-stranded human antibody domain targeting gp120 CD4i obtained by screening human antibody variable domain libraries using HIV-1 Env [77]. Later, M36 mutants with higher neutralizing activity were screened out. The antibody has a small molecular weight (15KDa) with a short half-life and is used to construct a bimolecular fusion protein [78]. 

Several other well-known CD4i-specific antibodies have been reported, include 17b [79,80], 48d, 47e, 412d, E51, 16C, 23e, 411g, C12, Sb1, X5 and m16 [81]. The N-terminus of the coreceptor CCR5 is sulfated tyrosine. Some CD4i-specific antibodies containing sulfated tyrosine, including E51 [82], 412d [83] and 47e [84], have shown stronger gp120-binding affinity than those without sulfated tyrosine. 

##### The gp120 CoRbs-Targeting Peptides

Two sulfated tyrosines (Tys173, Tys177) in the V2 loop of gp120 stabilize the intramolecular interaction of the V2 and V3 loops of gp120. Studies have found that the Tys177 sulfated peptide pV2α-Tys (amino acids 168–185) derived from the V2 loop can serve as a structural and functional mimetic of the N-terminus of CCR5, acting directly on the CoRbs on the gp120 V3 loop in a CD4-dependent manner, thereby blocking the binding of gp120 to the coreceptor and inhibiting HIV-1 infection [85]. pCCR5-Tys is a Tys-sulfated CCR5 N-terminal mimetic peptide that targets the CCR5 binding site on gp120 and effectively inhibits HIV-1 infection of CCR5^+^ cells [86]. It has been shown that pV2α-Tys competes with pCCR5-Tys for binding to gp120, establishing an intermolecular interaction with the CoRbs in the V3 loop [85]. Unlike pCCR5-Tys, pV2α-Tys is not restricted by HIV-1 coreceptor tropism and has a broad spectrum of anti-HIV-1 activity. Moreover, pE51 is a sulfated peptide derived from the CDR 3 region of the CD4i antibody E51, which inhibits the binding of HIV-1 to the CCR coreceptor in a CD4-independent manner [87]. However, the inhibitory activity of these peptides is at the µM level, and further optimization is needed.

#### 2.1.3. Antibodies or Recombinant Proteins Targeting gp120 Variable Loops and/or Glycan

##### Antibodies Targeting gp120 Variable Loops and/or Glycan

PG9 and PG16 are similarly potent mAbs isolated from HIV-1 patients. Compared to the earlier gp120-targeting bNAb 2G12 [88], both have higher neutralizing activity and broad spectrum in 162 different subtypes of HIV-1 pseudovirus neutralization experiments [89]. The neutralizing epitopes of PG9 and PG16 mainly depend on the specific position of the N-linked glycosylation on the gp120 V1-V3 loop and the glycan profile [90]. However, HIV-1 has gradually increased resistance to the bNAbs, such as 2G12, PG9 and PG16, during the virus evolution process [91,92]. Studies have shown that PG9 or PG16 and CD4-targeting monoclonal antibody ibalizumab (iMab) were constructed as bispecific antibodies, which not only have high inhibitory activity, but also effectively inhibited infection by PG9- and PG16-resistant strains [93]. Recent studies have found that 2G12, PG9 and PG16 can efficiently recognize HIV-1-infected CD4 T cells and induce CD4 T cell clearance by antibody-dependent complement-mediated lysis (ADCML) and antibody-dependent cell-mediated cytotoxicity (ADCC). Therefore, these bNAbs can be used in combinations to maximize the clearance of HIV-infected cells for HIV cure [94]. 

The four bNAbs, CH01 to CH04, are derived from the same clonal lineage isolated from HIV-1-infected individuals. They, which recognize conformational epitopes similar to PG9 and PG16 epitopes on gp120 V2/V3, can mainly neutralize tier 2, rather than tier 1, primary isolates. CH03 has the strongest neutralizing activity with mean and median IC50 of 2.4 and 0.46 μg/mL, respectively, against 91 HIV-1 primary isolates [95]. 

The PGT class of antibodies includes PGT121-123, PGT125-128, PGT130-131, PGT135-137, and PGT141-145 [96], which were isolated from elite controllers infected with HIV-1. These antibodies exhibited different HIV-1 neutralizing activities. PGT128 was the most potent bNAb; it had an IC50 of 0.02 μg/mL against 162 different subtypes of HIV-1 pseudovirus [96]. However, later studies found that circulating HIV-1 clade C in patients could escape the neutralization of PGT128 with N332 glycan specificity [97]. The crystal structure of the PGT128-gp120 complex indicates that PGT128 binds to a shorter β-sheet within the gp120 V3 loop. Since the V3 loop of gp120 is highly variable and easily masked by the HIV-1 Env trimer spike, the role of this antibody in humoral immunity may be limited [96].

10-1074 is a clonal variant of PGT 121 targeting the gp120 V3 loop isolated from a patient infected with subtype A HIV-1 [98,99]. The bNAb 10-1074 has neutralizing activity in vitro and in vivo [100]. Although a strain resistant to 10-1074 was found in phase I clinical trial [100], the combination of 10-1074 and 3BNC117 resulted in long-term effective inhibition of viral load in patients, and no resistant strains were found [101,102].

##### Recombinant Proteins Targeting gp120 Variable Loops and/or Glycan 

Some lectins have been found to interact with gp120 resulting in interference with the binding of gp120 to the CD4 receptor, thereby demonstrating anti-HIV-1 activity. However, their specific antiviral mechanisms of action are still unclear; nonetheless, such mechanisms may interact with glycans on gp120 to exert antiviral effects [103]. Cyanovirin-N (CV-N) is an 11KDa protein derived from cyanobacteria [104]. It irreversibly inactivates HIV-1/2 and SIV at low nM levels. Griffithsin (GRFT) is a 12.7KD protein derived from red algae [105], and it has been shown to inhibit HIV-1 infection and Env-mediated cell-cell fusion at the pM level. Deletion and rearrangement of glycosylation sites on gp120 may confer tolerance to CV-N and GRFT [106,107]. However, CV-N and GRFT can be amply expressed in *E. coli* with low cytotoxicity; therefore, both are expected to be further developed as microbicides. Recently, several legume lectins, including (ConBr) Canavalia brasiliensis lectin, (ConM) Canavalia maritima lectin, (DLasiL) Dioclea lasiocarpa lectin, (DScler L) Dioclea sclerocarpa lectin and (HHA) Hyppeastrum hybrid agglutinin all showed a low nM level of HIV-1 inhibitory activity and thus deserve further study [108].

## 3. Protein-and Peptide-Based HIV Entry Inhibitors Targeting gp41

It is quite effective to terminate the whole fusion process and inhibit virus invasion by blocking any single step in the fusion process. The transmembrane subunit gp41 is one of the most important targets for the design of fusion inhibitors because of its role in the viral-cell membrane fusion process. The gp41 extracellular domain consists of the N-terminal FP, the NHR, the loop region, the CHR, and the MPER (Figure 2A). The 6HB, a coiled-coil structure formed by three inner NHRs and three anti-parallel CHRs, is the core molecule gp41 function (Figure 1d) [9]. In addition, FP and MPER domains are also attractive targets, since several important anti-HIV peptides and neutralizing antibodies targeting these domains have been reported, as described below.

### 3.1. Antibodies or Recombinant Proteins Targeting gp41

#### 3.1.1. Antibodies and Recombinant Proteins Targeting gp41 NHR

##### The gp41 NHR-Targeting Antibodies

D5 is a human-derived scFv screened from a phage display library. It targets the NHR pocket-binding region of gp41 and blocks infection by different HIV isolates [109,110]. In addition, the attachment of D5 to cholesterol increases its membrane binding and antiviral activity, consistent with the results obtained by linking cholesterol to anti-HIV peptides and other mAbs [111,112,113]. MAb HK20 is isolated from HIV-infected individuals whose epitope covers the conserved hydrophobic pocket region in NHR. Surprisingly, the neutralization activity of HK20 on different target cells has varied greatly [114]. Subsequently, the crystal structures of HK20 and D5 combined with 5-helix were revealed. The combination of HK20 and D5 with NHR epitopes at different angles may allow HK20 to exhibit higher neutralizing activity and broad profile [115].

Other antibodies targeting NHR are rabbit scFv 8K8 and human Fab DN9, the epitopes of which partially overlap with the epitope of D5 and exhibit moderate neutralizing activity against different HIV strains [116]. In addition, the epitope of murine monoclonal antibody 1G12 may coincide with the target of T20 because it blocks the anti-fusion effect of T20 and binds to NHR-derived peptide [110].

##### The gp41 NHR-Targeting Peptides

C52L is a 55 amino acid recombinant peptide containing the entire T20 and C34 sequences with higher helicity and binding ability to NHR. It showed inhibitory activity similar to that of C34 in vitro, which was much better than T20. More importantly, C52L and its derivatives could be highly expressed in the *E. coli* system, up to 85mg-145mg per liter of culture. Although it needs further optimization, this is still exciting news for future low cost and large-scale production [117]. Our group has also designed a series of recombinant CHR-peptide fusion inhibitors, such as C66 (residues 610-675) and C72 (residues 610-681). They were not reported because of their low inhibitory activities (below that of T20). Interestingly, we found that the purification methods of the recombinant peptides were closely related to their anti-HIV activities. For example, CHR-peptide purified by GST-PreScission Protease system had the lowest activity, which may be caused by many reasons, such as the additional protease cleavage sites and the detergent in the elution buffer. 

TLT35 was designed by linking T20 to T1144 via a flexible linker [118]. This covalent attachment greatly enhances the enrichment of T20 and T1144 around the target, showing high activity and broad spectrum. Moreover, both T20 and T1144 fragments in TLT35 form a high α-helical structure, which enables TLT35 to be expressed in *E. coli* and has good resistance to protease. 

#### 3.1.2. Antibodies and Recombinant Proteins Targeting gp41 CHR

CHR-specific antibodies include murine monoclonal antibodies D40, D17, D50 [119] and FC-1, which exhibit different neutralizing activities against various HIV isolates [120]. In addition, human monoclonal antibodies HGF24 [114] and A2 were also found to specifically target the pocket-binding domain of gp41 CHR when CHRs maintain in the trimer conformation [121]. However, the neutralizing titers of these antibodies have not been reported, likely because of their lower neutralizing activity.

##### The gp41 CHR-Targeting Proteins

The recombinant protein NCCG-gp41 consists of N35 (residues 546-580)-N34 (residues 546-579)-SGGRGG-C28 (residues 628-655), wherein the 576-578 residues of N35 are replaced by CCG, resulting in the formation of a trimer [122]. The NCCG-gp41 trimer exhibits potent anti-HIV activity in vitro, and its IC50 is at low nM for HIV-1 Env-mediated cell-cell fusion.

Our groups have successfully designed and produced recombinant chimera protein HIV-1 fusion inhibitors, including N36Fd, N28Fd, and ccN28Fd. N28Fd and N36Fd were designed by fusing the NHR-peptides with a trimerized motif foldon (27 residues form T4 phage fibritin, highly hydrophilic and highly trimerized) which served to stabilize the chimeric trimer in physiological condition in order to allow the NHR-peptide part to efficiently interact with gp41 CHR [123] and were expressed as recombinant proteins in *E. coli.* N28Fd is both highly soluble and is able to form a trimeric conformation in neutral buffer. The viral inhibition activity of N28Fd is 135-fold more potent than that of N28 peptide, 15-fold more potent than that of N36 peptide and quite similar to T20 (low nM range). However, N28Fd is sensitive to proteolytic enzymes and low pH environments. The addition of two cysteines at its N-terminus constitutes ccN28Fd, which is resistant to heat, pepsin and proteinase K. ccN28Fd is approximately 20-fold more active than N28Fd in inhibiting HIV-1 IIIB and Bal infection [124]. In addition, since the inhibitory activity of ccN28Fd is not affected by semen and vaginal secretions, it also shows promise for the development of microbicides.

#### 3.1.3. Antibodies and Recombinant Proteins Targeting NHR/CHR Complex

A variety of antibodies targeting NHR/CHR complex were found, such as human monoclonal antibodies Fab-d [125], 50-69 [126], 126-7 [127], murine monoclonal antibodies NC-1 [128], 2G8 and 9F2 [129]. However, most of them cannot neutralize HIV at physiological temperature (37 °C) [125,128,129]. Interestingly, some antibodies inhibit HIV Env-mediated cell-cell membrane fusion at subtemperature (31.5 °C) [129]. It is believed that membrane fusion at physiological temperatures may be a rapid process and that steric hindrance hinders antibody binding, while molecular dynamics at subtemperature slows, prolonging the fusion process and allowing antibodies to be active [10,129,130].

We used the yeast two-hybrid technology to screen natural HIV protein inhibitors and finally obtained peptide P20, which is derived from human TNNI3K-like protein. Its derivative P20-A could inhibit a broad range of HIV-1 strains at low µM level, including T20-resistant strains. Interestingly, this peptide bound to 6HB and did not block the formation of 6HB. It suggested that the membrane fusion process was more complicated than we thought. The fusion did not complete immediately after 6HB formation. A series of previously unknown molecular events very likely takes place [131]. Miyauchi provided compelling evidence to show that HIV enters cells primarily by endocytosis [132], which may prove a similar view: that the virus and target cell membrane do not start to fuse immediately after gp41 changes conformation. Of course, this view is currently controversial.

In addition, using recombinant soluble gp41 (rsgp41) as bait, the human bone marrow cDNA library was screened by the yeast two-hybrid technique, and the endocytic protein POB1 binding to rsgp41 was identified. Further analysis revealed that C60 (residues 462-521), derived from the C-terminus of POB1 [133], is effective in inhibiting HIV-1 infection. Intensive research has found that it inhibits HIV-1-mediated membrane fusion, primarily by interacting with residues in the NHR exposed to the 6HB surface, but not blocking the formation of 6HB. In addition, POB1 mediates HIV-1 entry into epidermal cell A431, which lacks the CD4 receptor (CD4^−^), revealing that HIV-1 can infect CD4^−^ cells in a non-CD4-dependent manner [134]. Although C60 inhibits the activity of HIV-1 infection at low μM levels, it can be used as a probe to study the membrane fusion mechanism of HIV-1 and as a lead to develop HIV entry inhibitors targeting 6HB.

#### 3.1.4. Antibodies and Recombinant Proteins Targeting gp41 MPER 

##### The gp41 MPER-Targeting Antibodies

The HIV-1 gp41 MPER is a highly conserved region (Figure 2B), and a variety of human monoclonal antibodies have been found to target different epitopes in MPER and neutralize different subtypes of HIV-1. Therefore, MPER is also one of the important targets for HIV-1 vaccine design [135]. 2F5 neutralizes multiple HIV-1 laboratory-adapted and clinical strains and exerts antiviral activity by interacting with linear epitopes formed by the ELDKWA (residues 662-667) sequence in the gp41 MPER region [136]. 

Subsequently, another human monoclonal antibody, 4E10, was also obtained. It specifically recognizes the relatively conserved tryptophan-rich linear epitope (residues 671-676) located in the MPER. Although 4E10 has broad-spectrum neutralizing activity, it is exceptional for some strains of subtype B and subtype D. In addition, 2F5 and 4E10 have autoreactivity and can bind to the phospholipid bilayer of the MPER region [137], which is believed to cause some toxic side effects. 

10E8 is a bNAb derived from an HIV-1 patient whose epitope contains the residues of the transmembrane region (TM) and the entire MPER of gp41. Unlike 2F5 and 4E10, 10E8 has higher HIV-1 neutralizing activity and has no specific reactivity to self-antigens [138]. By identifying the hydrophobic region on 10E8 and optimizing its heavy and light chains, the 10E8 mutant 10E8v4 was successfully produced [139], which not only has higher solubility but also retains neutralization activity and profile similar to that of 10E8. Using CrossMab technology, some researchers have constructed a bispecific antibody library and screened out an excellent and broad-spectrum HIV-1 neutralizing antibody 10E8v2.0/iMab, which neutralizes 118 HIV-1 pseudoviruses with IC50 as low as 0.002 μg/mL [140]. In a humanized mouse model, 10E8V2.0/iMab has a positive effect on the prevention and treatment of HIV-1 infection. Studies have shown that the main reason for the high neutralizing activity of 10E8v2.0/iMab is the ibalizumab (iMab) component which can guide 10E8 v2.0 enrichment at the site where the fusion occurs, enabling the antibody to exert neutralizing activity more efficiently. 10E8.4/iMab with higher solubility and stability is obtained by introducing some hydrophilic amino acid mutations into 10E8v2.0/iMab [141]. Moreover, the in vitro and in vivo antiviral activity of 10E8.4/iMab was slightly higher than that of 10E8.2/iMab.

### 3.2. Peptides Targeting gp41

#### 3.2.1. Peptides Targeting gp41 FP

Kirchhoff’s group used a comprehensive peptide library generated from human hemofiltrate to make a large-scale screen, and they obtained peptide VIRIP derived from a human natural protein α1-antitrypsin. Instead of interacting with gp41 NHR or CHR, this peptide bound to gp41 FP. Its derivative, VIR-576, could inhibit infection of the diverse HIV-1 strains, including those resistant to T20 in vitro at low µM level [142]. Later, VIRIP was used to construct bispecific molecules with higher inhibitory activity [143]. It implies that other domains on gp41 may also serve might be designed as a drug targets for to development of novel HIV entry inhibitors. It also provided important details about the membrane fusion process, in particular, whether FP domain was exposed outside when gp120 made its conformational changes. VIRIP can also be used a molecule probe to study the function of the gp41 FP in membrane fusion process.

At present, it is reported that co-infection of GBV-C (a lymphotropic virus that replicates in primary T and B lymphocytes) with HIV-1 is beneficial to patients with HIV-1 disease [144,145,146], but different views prevail [147]. For safety reasons, the researchers used GBV-C proteins, such as E1 and E2, to study their anti-HIV-1 activity, and they found that some peptides derived from E1 or E2 may inhibit HIV-1 infection by interacting with HIV-1 FP [148,149,150,151,152,153]. The 18-amino acid peptide E1P47 was screened from the E1 protein overlapping peptide library, and it has a broad spectrum of HIV-1 inhibitory activity [154]. Peptide P6-2 is derived from the E2 protein, and its inhibitory activity against the original isolate is comparable to that of VIR-576 [151].

#### 3.2.2. Peptides Targeting gp41 NHR

**Figure 3:**
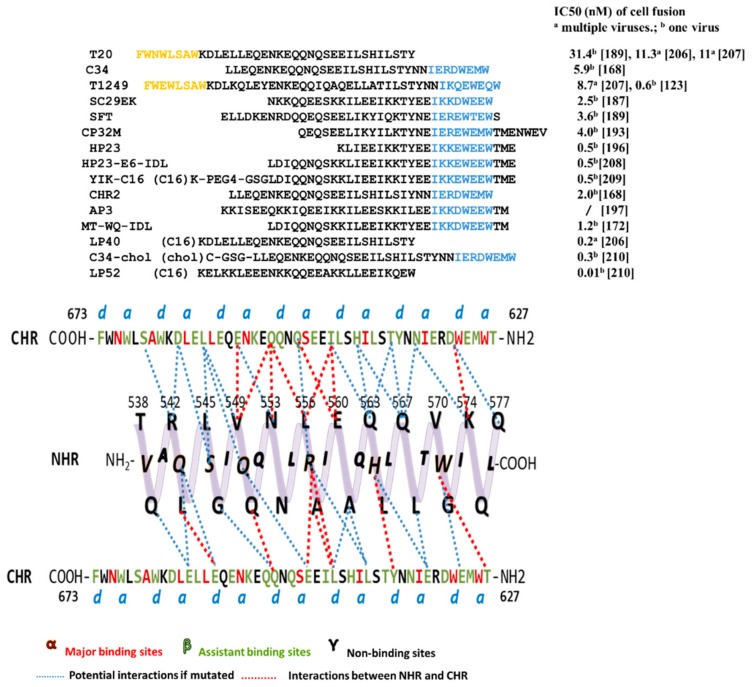
Tripartite model of gp41 N-terminal heptad repeats (NHR) with side view and C-terminal heptad repeats (CHR)-derived peptides. Above, the sequences of CHR-peptides targeting NHR. Residues represent pocket-binding domain and lipid- binding domain marked in blue and orange, respectively. Below, residues of CHR are divided into three groups: major binding sites α, assistant binding sites β, and non-binding sites γ. The potential interaction, or interaction, between CHR and NHR is shown in blue or red dashed lines.

For many enveloped viruses, 6HB is an important target for the design of fusion inhibitors [155,156,157,158]. In 1997, using crystallography, Chan’s group proved that NHR and CHR strongly interacted with each other to form the coiled-coil six helix bundle in vitro. This detailed structure explained how NHR- and CHR-derived peptides could inhibit viral-cell fusion effectively. More specifically, the synthetic peptide bound to the fusion intermediates and occupied the position, preventing natural NHR/CHR to form the active 6HB, followed by terminating membrane fusion. This laid the foundation for the design of fusion inhibitors [159,160,161,162,163,164,165]. Two representative peptides, C34 (CHR-peptide) and N36 (NHR-peptide) [166], form a six-helix complex in vitro with the N36 trimer as the core and the three C34 peptides in anti-parallel, similar to native 6HB. Since then, this in vitro six-helix complex has been thought to mimic native 6HB and is therefore used to screen for fusion inhibitors that target NHR or CHR [167]. Although a number of peptides have been designed based on this classical in vitro 6HB model, it only considers the position and orientation of the CHR backbone, thus bringing limitations to the optimization of inhibitors. A modified new model NHR-CHR-NHR was proposed, in which more CHR residues were found (assistant binding sites, β) to interact with NHR (Figure 3). Some CHR-peptides based on the new model show various HIV-1 inhibitory activities, demonstrating the importance of the assistant binding sites [168]. Currently, most peptide fusion inhibitors are derived from gp41 NHR and CHR, especially the CHR domain. Most of the amino acids of NHR are very important for viral infection, and it is a popular research target for CHR-peptides [169]. In fact, CHR-peptides are more effective than other peptides in HIV inhibition. Here is a summary of the amino acid sequences of CHR-peptides and their inhibition of cell-cell fusion activity (Figure 3). However, we should also consider their biochemical properties [170,171], such as solubility and stability [172]. 

##### The First Generation CHR-Peptides

The first generation CHR-peptides consist of three peptides derived from the HIV-1 gp41 CHR domain, including SJ-2176 (residues 630-659), DP-178, which was named T20 later (residues 638-673), and C34 (residues 628-661), which were reported by Jiang et al. at the New York Blood Center in 1993 [163,164], by Wild et al. at the Duke University 1994 [165], and by Lu et al. at MIT in 1995 [166], respectively. New York Blood Center filed the patent on SJ-2176 (US Patent 5,444,044) on March 26, 1992 (issued on August 22, 1995), while Duke University filed the patent on T20 (US Patent 5,464,933) on June 7, 1993 (issued on November 7, 1995). The group at Duke University established a pharmaceutical company named Trimeris, Inc. to develop T20 as a fusion inhibitor-based anti-HIV drug. As T20 has 22 amino acids overlapping SJ-2176, Trimeris had to license New York Blood Center’s patent in 1997 for the development of T20.

T20 (enfuvirtide) was approved by the U.S. FDA in 2003 as the first HIV fusion inhibitor-based anti-HIV drug for clinical use to treat HIV-infected patients who have failed to response to current ARDs [173,174,175]. However, its clinical application is limited because of its low potency (about 10-fold less potent than C34) and short half-life (about 2 h), thus requiring subcutaneous injections twice daily at a dose of 90 mg and causing severe injection site reactions [176,177]. Our previous studies have shown that unlike C34 that contains a pocket-binding sequence (PBD), which is critical for the interaction between a CHR-peptide and viral gp41 NHR to form stable 6HB and inhibition of HIV fusion, T20 lacks PBD and thus cannot interact with viral gp41 NHR as effectively as C34 to block HIV infection [178,179]. However, T20 contains a tryptophan-rich motif (TRM) or lipid-binding region (LBD). We thus believed that T20 needs the LBD to interact with target cell membrane to enhance its interaction with the viral NHR domain [178,179]. Zhang et al. have shown that T20 is able to interact with N39 peptide, which contains partial sequences of the NHR domain and the fusion peptide proximal region (FPPR), the downstream region of the C-terminus of the FP, to form 6HB [180]. Most recently, we have demonstrated that T20 can inhibit the formation of the native 6HB on HIV-1-infected cells, but only in the early, not the late stage of fusion, and the interaction with FPPR may play an important role in its inhibitory activity. Based on this newly clarified mechanism of action of T20, we designed a new peptide, T20-SF, by adding an additional TRM to the C-terminus of T20 and found that T20-SF exhibited significantly improved anti-HIV-1 activity because it targets the triple sites in gp41, including NHR, FPPR, and FP [181]. 

##### The Second Generation CHR-Peptides

The second generation CHR-peptides are the mutant peptides of T20 or C34 with anti-HIV-1 activity about 10- to 20-fold more potent than T20, such as SC34 and its analogs, T1249 and T1144, sifuvirtide and albuvirtide.

A series of CHR-peptide mutants has been designed to improve their solubility, NHR-binding affinity, antiviral potency, or half-lives [182,183,184]. Fujii’s group modified the C34-like peptide to increase its solubility and enhance its interaction with NHR by introducing EE-KK double salt-bridge. The first designed peptides, SC34 and SC35EK, showed activity similar to that of C34, while they interacted with gp41 NHR more strongly and were more soluble than C34, thus being better drug candidates for further development [182]. SC35EK was further shortened to SC29EK and kept similar HIV-1 inhibitory potency [185,186,187]. The same strategy was applied to T20 and resulted in T20EK, with ~10-fold potent increase compared with T20. It is also effective against T20-resistant HIV-1 isolates [186].

T1249 and T1144 were developed by Trimeris, Inc., in a hope to replace T20 [179,182,183,184]. T1249 contains C34’s PBD and T20’s LBD. Although T1249 had enhanced anti-HIV-1 activity against T20-resistant HIV-1 strains, its Phase II clinical trial was terminated because of the reports of side effects [161]. T1144 was designed based on the sequence of C38 peptide (residues 626-663). In vitro, it shows stronger antiviral activity than T1249 against infection of divergent HIV-1 strains, including those resistant to T20 [188]. In particular, it maintains a stable α-helic trimeric structure (up to 97%) in neutral buffer. These characteristics contribute to its enhanced anti-HIV potency and stability [182]. However, some studies have proved that certain CHR-peptides with high helicity (100%) and stable binding with NHR (Tm value > 100 °C) show little anti-HIV activity [184]. Therefore, the appropriate affinity with NHR and the proper helicity may lead to the highest anti-HIV activity.

Sifuvirtide is a novel peptide inhibitor developed by Fusogen, Inc. [189]. Its sequence is derived from C34 with some mutations, and the spiral property is enhanced. Similar to C34, sifuvirtide strongly bound to NHR-peptide and significantly inhibited 6HB formation [190]. It also has a more potent anti-HIV activity in vitro than T20 [189], especially to T20-resistant HIV-1 strains. Sifuvirtide has successfully passed Phase I and II clinical trials in China. Also, the efficacy of sifuvirtide in monotherapy at 20 mg once daily is equivalent to that of enfuvirtide at 90 mg twice daily (http://www.fusogen.com). However, its further development has been terminated with unknown reasons.

According to the crystal structure of the complex formed by C34 and NHR-peptide, FB006 was designed by substituting three residues in C34 which were not bound to the NHR-peptide with Lys and Glu. Conventional peptide inhibitors are susceptible to protease degradation and therefore have a short half-life in vivo, but their binding to serum albumin prolongs their half-life in vivo [191]. Conserved Cys residues occur in different species of albumin (Cys34 in humans), and Cys only has the free sulfhydryl group. In order to bind the peptide to the thiol group in albumin, different residues of FB006 were modified with maleimidopropionic acid (MPA) to obtain corresponding modified peptides. These modified peptides were bound to human serum albumin (HSA) and tested for their anti-HIV activity in vitro. FB006M obtained by chemical modification of the 13th residue of FB006 with MPA is not immunogenic. The half-life of FB006M in rhesus monkeys is more than 100h, which is about 10-fold longer than that of FB006 [192]. In clinical trials, FB006 (albuvirtide) administered intravenously with current ARDs once a week could effectively inhibit HIV-1 infection and reduce viral load. Therefore, it was approved by the China Food and Drug Administration (CFDA) for clinical use in 2018.

##### The Third Generation CHR-Peptides

The third generation CHR-peptides are the modified CHR-peptides with anti-HIV-1 activity more than 50-fold more potent than T20, including CHR-peptides containing MT-hook or IDL anchor, and lipopeptides.

##### CHR-Peptides Containing MT-Hook

He et al. have shown that the peptide CP32 (residues 621-652) exhibited more potent anti-HIV-1 activity than T20 and C34. Its derivative, CP32M, inhibits not only T20-resistant strains, but also C34-resistant strains [193,194], providing a novel paradigm for the development of a new generation of entry inhibitors. Analysis of the crystal structure of 6HB formed by CP32 or CP32M, and NHR-peptide DP107 (T21) revealed that Met-626 and Thr-627 in CP32 can form a hook-like structure (called MT-hook), which can stabilize the interaction of CHR-peptide and the pocket-forming domain (PFD) of NHR, and significantly increase the inhibitory activity of CHR-peptide [190]. Therefore, MT-hook is widely used in the design of CHR-peptides. HP23, a 23-residue peptide, which contains MT-hook and PBD, has high binding stability and antiviral activity, including T20-resistant, or MT-SC22E-resistant strains [195,196]. Subsequently, the short peptide 2P23 containing the MT-hook and the HIV-2 sequence was designed. The highly stable helical peptide 2P23 has broad-spectrum inhibitory activity against different subtypes of HIV-1, including T20-resistant strains and HP23-resistant strains [195]. However, compared to HP23, 2P23 has low HIV-1 inhibitory activity. The CHR-peptides derived from gp41 CHR contain some natural sequences and may, therefore, crossreact with pre-existing antibodies in the serum of HIV patients. In order to avoid this phenomenon, we designed artificial peptides AP1 and AP2 and added MT-hook at the N-terminus of AP2 to stabilize the interaction with NHR to obtain AP3, which can fold into a single helix to interact with gp41 NHR to prevent 6HB formation [197]. In addition, AP3 is superior to T20, AP1 and AP2 in antiviral activity, antiviral profile and pharmacological properties. Surprisingly, pre-existing antibodies in patients not only fail to recognize AP3, but also enhance its anti-HIV activity. This mechanism is still worth exploring.

##### CHR-Peptides Containing IDL Anchor

Using a computer model to analyze the crystal structure of the NHR trimer, we found a shallow pocket at the N-terminus of the NHR (L544-V549), which was named the N-terminal hydrophobic pocket (NTHP). It consists of Leu544 and Ile548 of one NHR helix and Leu545 and Val549 residues of another NHR helix [172]. These residues are highly conserved among different HIV-1 strains, and we hypothesized that enhancing the binding of the CHR-peptide to NHR NTHP could increase its inhibitory activity. Therefore, we designed a series of long-chain hydrophobic residues at the C-terminus of CHR-peptide WQ (residues 628-653) to target NTHP. As expected, the newly designed peptide WQ-IDL was more active than T20 in inhibiting HIV-1 IIIB and Bal infection. Introducing the MT-hook at the N-terminus of WQ-IDL, we found that the activity of MT-WQ-IDL was further improved [172]. It may be that MT and IDL enhance the binding of WQ peptide to NHR PFD and NTHP, respectively [172]. Peptide WQ is also termed peptide CP. CP-IDL was coupled to N36 or N43 using the "SGGRGG" linker to form an N36-L6-CP-IDL or N43-L6-CP-IDL complex. The crystal structure indicates that the IDL tail can form two different conformations. One is alpha-helix (N36), and the other is a hook-like structure (N43), which exhibits a broader interaction with NHR [198].

##### Lipidated CHR-Peptides

It is widely believed that lipids in lipopeptides are able to anchor peptides to the cell membrane, resulting in elevated concentrations of peptides at the fusion site [199,200,201,202]. However, Klug et al. proposed a different mechanism of the sphinganine lipidated peptides, in which sphinganine and PBDK peptide, a 15-mer peptide derived from the pocket-binding domain of the gp41 CHR region, jointly disrupt early and late events of membrane fusion [203]. Furthermore, lipopeptides exhibited prolonged half-life in vivo than unconjugated peptides, possibly because they can reversibly bind to proteins in serum [163,188,204]. He et al. modified HP23 and 2P23 with different lipids (e.g., fatty acids, cholesterol, sphingolipids). HP23 and 2P23 were conjugated to palmitic acid (C16), respectively, and the MT-linked methionine in HP23 was replaced with leucine to construct LP-11 and LP-19, which mainly targeted the PFD of gp41 NHR and inhibited different HIV-1 clinical isolates, including T20-resistant strains and HP23 or 2P23-resistant strains. In addition, LP-19 can effectively inhibit HIV-2 and SIV infection [199,205]. The same team then designed a series of lipopeptides, for example, to design LP40 by replacing the T20 C-terminal tryptophan-rich region (TRM) with C16. Interestingly, LP40 inhibits cell-cell fusion and pseudovirus entry activity complementary to LP-11, thus having a synergistic antiviral effect [206]. Similar to the LP40 design strategy, LP46 was constructed by replacing the T1249 C-terminal TRM with C16. LP46 has higher inhibitory activity than LP40, with IC50 values down to pM levels, and exhibits synergy with LP40 [207]. We previously designed the HP23-E6-IDL peptide and later introduced a single amino acid mutation and added palmitic acid to obtain YIK-C16 lipopeptide, which exhibits high antiviral activity and a long half-life [208,209]. LP52 is a 28 amino acid lipopeptide designed based on the analysis of the structure-activity relationship of LP40 and LP46. It does not contain PBD and TRM, but its C-terminus is coupled to C16. LP52 has a high gene barrier and a long half-life. It also inhibits a large number of HIV-1 isolates at low pM levels and also has high activity against T20-resistant strains [210]. Most of these lipopeptides are palmitic acid modified with existing CHR-peptides containing or lacking PBD. Lipopeptides have better activity, half-life and genetic barrier than their precursor peptides, but, as a consequence, they cost more. A large number of resistant strains against CHR-peptides have emerged, greatly limiting the use of most CHR-peptides. Whether resistance to lipopeptides will occur in the future is still the focus of our attention. 

Covalent binding of cholesterol to C34 obtained C34-chol (DS007). C34-chol has high affinity to lipid rafts of cell membranes and inhibits the formation of 6HB during viral infection. The activity of C34-chol to inhibit multiple HIV-1 isolates in vitro is about 25- to 100-fold that of C34 activity [211], indicating that linking cholesterol to HR-derived peptide inhibitors is an effective strategy for increasing antiviral activity. Furthermore, this strategy can also be applied to enhance the antiviral activity of fusion inhibitory peptides against enveloped viruses, such as influenza virus, suggesting that the role of cholesterol in the design of HIV-1 entry inhibitors deserves more attention. In contrast, palmitic acid used in lipids to enhance the interaction of peptides with cell membranes is more convenient and economical than cholesterol and sphingolipids [199]. In addition to enhancing the binding to lipids, palmitic acid can also bind to human serum albumin, which can reduce the rate of glomerular elimination [212] and prolong the half-life of the drug in vivo.

Lipid rafts are thought to be the hallmark of HIV-1 budding and entry into T cells and macrophages [190,213,214], and many glycosylphosphatidylinositol (GPI)-anchored proteins are localized in lipid rafts. GPI-C34 (C34 anchored with GPI) has been shown to bind to lipid rafts via GPI anchors and broadly inhibit HIV-1 infection [215]. This again proves that the inclusion of substances capable of binding to lipid membranes in HIV inhibitors is an effective way to increase the activity of inhibitors.

#### 3.2.3. Peptides Targeting gp41 CHR

**Figure 4:**
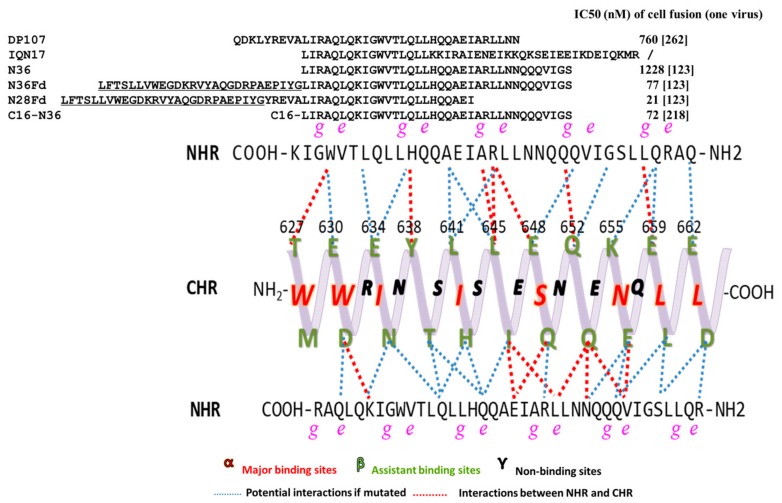
Tripartite model of gp41 C-terminal heptad repeats (CHR) with side view and N-terminal heptad repeats (NHR)-derived peptides. Above, the sequences of peptides targeting CHR. The sequences of Fd are underlined. Below, residues of CHR are divided into three groups: major binding sites α, assistant binding sites β, and nonbinding sites γ. The interaction, or potential interaction, between CHR and NHR is shown in red or blue dashed lines.

The focus on developing CHR-peptides arises from the early discovery of some NHR-derived peptides that interact with CHR to inhibit the formation of homologous 6HB, but they have lower inhibitory activity and are unstable. Here is a summary of the amino acid sequences of NHR-peptides and their inhibition of cell-cell fusion activity (Figure 4).

N36, an NHR-peptide with 36-amino acid residues derived from the HIV-1 gp41 NHR, can interact with a CHR-peptide derived from the gp41 CHR to form 6HB in vitro [216]. Theoretically, it should have inhibitory activity against HIV-1 infection since it can interact with the viral gp41 NHR to form heterologous 6HB, thus blocking fusion between viral and cell membranes. However, N36 and other NHR-derived NHR-peptides have the tendency to aggregate in neutral buffer because of its strong hydrophobicity, thus exhibiting very low viral fusion inhibitory activity (about hundreds of folds lower than that of a CHR-peptide) [178]. The peptide N36^Mut(e,g)^ was designed by replacing the hydrophobic residues at the e, g positions of N36 with hydrophilic residues so that this NHR-peptide cannot interact with CHR to form 6HB, while it remains soluble in physiological solutions or PBS. N36^Mut(e,g)^ showed HIV-1 inhibitory activity at sub-micromolar level. This peptide may interact with the viral gp41 NHR to form non-functional heterologous NHR trimer, resulting in the inhibition of fusion between the viral and target cell membranes [217]. Shai and colleagues have shown that N27, a 27-residue NHR-peptide with no PBD, has no significant inhibitory activity in the cell-cell fusion; but it became very active (IC50=10 nM) when palmitic acid (C16) was conjugated to its N terminus (C16-N27), but not C terminus (N27-C16). Interestingly, N27^Mut(e,g)^, which has the similar mutations as N36^Mut(e,g)^, exhibited no cell fusion inhibitory activity either. C16 conjugated at its N-terminus, i.e., C16-N27^Mut(e,g)^, showed some inhibitory against HIV-1 Env-mediated cell-cell fusion, but it is about 63-fold lower than that of C16-N27 [202], suggesting that N27 mainly targets the CHR rather than the internal NHR coiled-coil. This group has also demonstrated that conjugation of C16 to either N- or C-terminus of N36 results in increase of membrane fusion inhibitory activity, while the inhibitory activity of N36^Mut(e,g)^-C16 is about 17-fold stronger than that of C16-N36^Mut(e,g)^ [202,218], suggesting a planar orientation of the peptide and the endogenous NHR region on the cell membrane. suggesting a planar orientation of the peptides as well as the endogenous NHR region on the cell membrane.

Efforts have focused on how to enhance NHR-peptide solubility and maintain its natural trimer structure. Kim added a highly trimerized model peptide, such as GCN4 sequence, IQ, IZ, and others, to N17 to form recombinant IQN17 and CC-IZN17. They were all highly soluble, formed trimer structure and increased anti-HIV activity from μM to low nM range [159]. 

Mutated NHR-peptides, however, are not quite stable compared to CHR-peptide; therefore, they cannot maintain their antiviral activity for very long. Nevertheless, the study of NHR-peptide fusion inhibitors is still attractive because these inhibitors have the advantage of targeting gp41 CHR, which may avoid cross-drug resistance with currently used fusion inhibitor T20 and other CHR-peptide fusion inhibitors under development [161]. 

## 4. Protein-Based HIV Entry Inhibitors Targeting both gp120 and gp41

In vitro data show that inhibitors targeting gp120 and gp41 have potent antiviral activity. The discovery of some antibodies targeting both gp120 and gp41 indicates that the N-terminal FP of gp41 can be exposed in solution and that it is a new target. The residues involved in the gp41-gp120 interface in the gp120 core were also resolved [7]. Furthermore, recombinant proteins with multiple targets have improved antiviral activity and may be less susceptible to inducing resistant strains and thus may be an attractive research direction. 

### 4.1. Antibodies Targeting both gp120 and gp41

8ANC195, a bNAb isolated from HIV-1 patients; it neutralizes 118 HIV-1 tier 2 isolates with an IC50 of 0.5-12.1 μg/mL [19]. Its epitopes include Asn 234 and Asn 276 on gp120, the inner region of gp120, loop D and loop V5 and may be involved in Asn637 and its adjacent amino acids on gp41 [219]. Since the epitope spans gp120 and gp41 in the native Env trimer, it becomes the first bNAb derived from humans acting on two subunits of Env.

35O22 is a bNAb produced primarily in natural infections and targets the conserved interface of gp120-gp41. It recognizes the glycosylation sites N88A, N230A, N241A and N625A on the HIV-1 JRCSF strain Env and has higher neutralizing activity than PGT121 and VRC01. After gp120-sCD4 complex causes a change in the conformation of gp41, studies have shown that 35O22 binds to the exposed epitope at the interface of gp120-gp41 to exert neutralizing activity. Therefore, it does not compete with other bNAbs that target gp120 to bind viral particles [220]. Many HIV-1-infected individuals have antibodies similar to 35O22, which opens up the possibility of making vaccines similar to this antibody. Furthermore, the construction of immunogens that are structurally similar to natural Env trimers may have an important role in inducing such antibodies.

The bNAb VRC34.01 was derived from HIV-1 chronically infected individuals, inhibiting the conformational changes of HIV-1 gp120 and gp41 and blocking viral entry. Like 35O22, the epitope of VRC34.01 contains N88 on gp120, but it also targets the N-terminus of gp41 FP. Its neutralization profile is limited with a neutralization rate of 49% for 208 different HIV-1 strains at IC50 below 50 μg/mL. Mutations in gp120 N88 and FP cause severe resistance to VRC34.01 [221]. However, molecular dynamics indicate that the N-terminus of FP can be exposed to solution, which means that the discovery of this antibody suggests that FP is also an important antibody recognition epitope.

PGT151 is a bNAb isolated from elite controllers infected with HIV-1 [222]. It targets the gp120-gp41 interface and FP of gp41 [3,223] and stabilizes the highly unstable Env trimer structure. Compared to 8ANC195, 35O22 and VRC34.01, PGT151 has a broader neutralization profile with a neutralization rate of 68% for 208 HIV-1 at IC50 <50 μg/mL [221].

bNAb ACS202, which targets the interface of gp120-gp41, was isolated from an elite controller infected with HIV-1. It interacts with gp120 residues and glycans (especially N88) and with gp41 FP and FPPR [224]. It only neutralized 45% of the 75 different subtypes of HIV-1 strains with a median IC50 value of 0.142 μg/mL. Although the neutralizing activity and range are not prominent, the discovery of this antibody revealed that conserved FP is a target for bNAb.

mAbs 3BC315 and 3BC176 were isolated from the same patient with moderate broad-spectrum, neutralizing activity and similar epitopes [225]. 3BC315/3BC176 targets the interface of gp120-gp41, and its mechanism of neutralizing activity may one that destroys the function and integrity of Env trimer, causing the shedding of gp120 [226]. 3BC315 / 3BC176 is sensitive to antibody-resistant strains targeting gp120 CD4bs in the same patient; therefore, the combination can broaden its neutralization profile.

mAb CAP248-2B was recently isolated from HIV-1 patients and targeted to the gp120-gp41 contact surface. Its epitope contains the C-terminus of gp120 and partially overlaps with the epitopes of PGT151, VRC34, 35O22 and 3BC315 on gp41 [227]. However, the neutralization range of CAP248-2B is limited, and some strains of gp120 C-terminal mutation can escape its neutralization.

mAb M43 was obtained by screening phage antibody Fab library and gp 41 specific mAb library using the competitive antigen panning (CAP) methodology [228]. The conformational epitope recognized by M43 contains CD4bs on gp120 and N-trimer structure on gp41. However, the neutralization activity and neutralization range may limit its application.

### 4.2. Proteins Targeting both gp120 and gp41 

2DLT is a bivalent chimeric protein constructed by fusing T1144 with the first two extracellular domains of CD4 (D1D2). Unlike most entry inhibitors, it triggers early exposure of gp41 prior to viral fusion and rapidly inactivates viral particles [229]. Binding of D1D2 to CD4bs on gp120 causes a conformational change in gp120 and the exposed gp41, which results in the formation of a prehairpin fusion intermediate (PFI). T1144 then binds to the exposed PFI and causes the cell-free virus to rapidly “inactivate”. The EC50 of 2DLT inactivated different HIV-1 isolates ranging from 17.3 to 78.6 nM, making it more effective than T20 and T1144 (EC50 >500 nM). What’s more, 2DLT can be produced in large quantities in *E. coli* and exhibit synergy with other antiviral drugs, rather than mediating HIV-1 infection like sCD4 [229,230]. This chimeric protein can serve as a dual barrier against HIV-1 infection. First, HIV-1 is "inactivated" and unable to infect target cells. Second, if the virus evades the first attack of 2DLT, T1144 in 2DLT can inhibit viral-cell fusion as a fusion inhibitor. 

sCD4-FIT45 is a bispecific protein in which sCD4 is covalently linked to a 45 amino acid CHR-peptide T45 [231] targeting gp41 NHR. Similar to 2DLT, sCD4 in sCD4-FIT45 interacts with gp120 to expose gp41 NHR, followed by T45 targeting NHR, inactivating cell-free virus [232]. Unlike sCD4, the bispecific protein sCD4-FIT45 does not cause HIV-1 Env-mediated CD4^−^CCR5^+^ cell infection [233], probably owing to the important role of FIT45.

Griff37 consists of a GRFT (griffithsin) targeting gp120 and a peptide C37 (3 more amino acids at the N-terminus of C34) targeting gp41 NHR. It inhibits cell-cell fusion and HIV-1 infection at mid-nanomolar and mid-picomolar levels, respectively, and its inhibitory activity is more effective than that of GRFT or C37 or a combination of both [234]. This further demonstrates that GRFT activity can be increased by coupling drugs of different targets. In addition, since the resistant strain against GRFT has emerged [106], Griff37 has dual targeting and may increase the genetic barrier. 

C37CD4M33C1F23 is a dual-targeted recombinant protein formed by covalent attachment of peptide C37 targeting gp41 NHR to CD4M33C1F23 (with Cys at position 1 and Phe at position 23) targeting gp120 [234]. The recombinant protein inhibited the activity of the CCR5^-^tropic fusion assay significantly higher than the individual components, although the difference was not significant in the inhibition of the CXCR4^-^tropic fusion assay. However, because of the simplicity of preparation of the recombinant protein, this strategy can be used as a reference to guide the synthesis of other recombinant proteins.

## 5. Conclusions

Since the first peptide-based HIV entry inhibitor, enfuvirtide, was approved for clinical use by the U.S. FDA in 2003, about nine series of protein- and peptide-based HIV entry inhibitors have been developed in pre-clinical and clinical studies (Table 1). Different from most of the current ARDs that must enter the host cells to inhibit HIV replication, these entry inhibitors block HIV entry into the host cells by targeting the site(s) on the viral surface proteins, including the CD4bs, CoRbs, variable loop region, and glycans on gp120, or FP, NHR, or CHR, 6HB, and MPER on gp41. Therefore, these big molecules (proteins or peptides) without cell-penetrating ability can act on the surface of cells to block the virus entry into the cell or transmission between the cells. They are expected to have no adverse effects on the functions of the intracellular proteins. High specificity, potency and safety are the advantages of the protein- and peptide-based HIV entry inhibitors, while high cost of production and lack of oral availability are their disadvantages, compared with the current ARDs. However, these may not be a concern if they are used to treat the patients at the early stage of highly pathogenic virus infection with high mortality, as a short-term (1-2 weeks) injection of a protein- or peptide-based virus entry inhibitor at high dosage is expected to save the patients’ lives. 

Currently, a number of protein-based HIV entry inhibitors, particularly the gp120- or gp41-specific neutralizing antibodies, with high specificity, efficacy and safety, as well as long half-life in vivo have been reported [14,138,235,236,237]. Some of them are already undergoing clinical trials [22,23,138,141]. Unlike the protein-based HIV entry inhibitors that must be stored and transported at low temperature, the peptide-based HIV entry inhibitors can be stored and transported at normal temperature. However, the peptide-based HIV entry inhibitors are generally susceptible to protease degradation, thus having shorter half-life in vivo [238,239,240]. Some studies have attempted to use *E. coli* expression systems or conjugation techniques to reduce the cost of inhibitors or enhance stability [192,211,241]. In addition, the emergence of drug-resistant strains is also an urgent problem to be solved. Therefore, optimization of the sequence and structure of the peptide-based HIV inhibitors for the purpose of prolonging their half-life, enhancing their anti-HIV potency, improving their druggability, and delaying the emergence of drug-resistant strains by combinational use of two or more HIV entry inhibitors with different targets are essential for their future development.

## Figures and Tables

**Table 1:** Protein-and peptide-based HIV entry inhibitors targeting gp120 or gp41.

Inhibitor	IC_50_		No. of Isolates	Target Cells	Animal Trials	Clinical Phase	Reference
	μg/mL	nM					
Inhibitors targeting gp120 CD4bs							
Proteins							
D1D2/mD1.2		35/19 ^a^	13	HOS			[57]
PRO-542	7 ^a^		28	PBMC	mice	Terminated	[53,54]
6Dm2m		0.32 ^a^	41	TZM-bl			[58]
VRC01	0.33 ^b^		190	TZM-bl	NHP, mice	Ⅰ	[14,235,236]
b12	1.79 ^b^		190	TZM-bl	NHP, mice		[14,36,242]
NIH45-46/45-46m2	0.41 ^b^/0.028 ^b^		65/118	TZM-bl	mice		[20,21,235]
N6	0.038 ^c^		181	TZM-bl	NHP	Ⅰ	[22,23]
3BNC117	0.11 ^c^		180	TZM-bl	NHP, mice	Ⅱ	[24,25,28,138]
PGV04	0.19 ^b^		178	TZM-bl			[32]
Peptides							
CD4M33		424.7 ^a^	4	PBMC			[65]
M48U1		0.71 ^a^	4	GHOST	NHP		[68,70]
Inhibitors targeting gp120 CoRbs							
Proteins							
17b	NA		7	Cf2Th			[82]
E51	NA		7	Cf2Th			[82]
M36/M36.4		17 ^b^/7.3 ^b^	13	HOS			[78]
Peptides							
pV2α-Tys		<50000 ^a^	12	primary human CD4+ T			[85]
pCCR5-Tys		>50000 ^a^	12	primary human CD4+ T			[85]
Inhibitors targeting gp120 variable loops or glycans							
Proteins							
F105		NA			NHP	Ⅰ	[243]
2G12	2.43 ^c^		162	U87	NHP/mice	Ⅱ	[89,244,245,246]
PG9/ PG16	0.2/0.15 ^c^		177	TZM-bl	NHP/mice		[138,236,247]
CH03	0.46 ^c^		91	TZM-bl			[95]
10-1074	0.18 ^d^		306	TZM-bl	NHP/mice	Ⅰ	[21,100,247,248]
PGT121	10.49 ^c^		162	U87	NHP/mice	Ⅰ	[23,96,249,250]
PGT128	2.73 ^c^		162	U87	NHP/mice		[96,251,252]
CV-N		8.2 ^a^	20	TZM-bl			[253]
GRFT		1.0 ^a^	18	TZM-bl			[253]
Inhibitors targeting gp41 FP							
Peptides							
VIR-576		NA		PBMC		Terminated	[142]
E1P47		8200 ^a^	6	TZM-bl			[154]
Inhibitors targeting gp41 NHR							
Proteins							
D5		554.4 ^a^	5	U87			[109]
HK20	25.2 ^a^		18	HOS			[115]
8K8		253.6 ^a^	11	U87			[116]
C52L		21.5 ^a^	8	PBMC	NHP/mice		[117,254,255]
TLT35		5.7 ^a^	11	PBMC, MT-2			[118]
HR212		2.8	1	GHOST			[256]
Peptides							
SJ-2176		101 ^a^	1	MT-2			[163,164]
T20		29.45 ^a^	25	TZM-bl	NHP, mice	Approved	[165]
C34		12.5 ^a^	10	MT-2, M7			[166]
SFT		50 ^a^	6	PBMC	NHP, mice	Terminated	[189,257,258]
SC29EK		9.6 ^a^	7	HeLa			[185]
T1249		3.44 ^a^	25	TZM-bl	NHP	Terminated	[259,260]
T1144		13.9 ^a^	11	PBMC, MT-2			[118,184]
FB006M		2.7 ^a^	8	PBMC	NHP, mice	Approved	[192]
CP32M		65 ^a^	10	PBMC			[193]
HP23		4.7 ^a^	29	TZM-bl			[195]
AP3		19 ^a^	8	MT-2, M7			[197]
HP23-E6-IDL		0.75 ^a^	12	MT-2, M7			[208]
CHR2		2.4 ^a^	22	MT-2, M7			[168]
YIK-C16		0.09 ^a^	18	MT-2, M7			[209]
MT-WQ-IDL		2.7 ^a^	24	MT-2, M7			[172]
LP-11		0.83 ^a^	25	TZM-bl			[206]
LP40		4.29 ^a^	25	TZM-bl			[206]
LP46		0.08 ^a^	25	TZM-bl			[210]
LP52		0.017 ^a^	35	TZM-bl			[210]
C34-chol		15.5 ^a^	6	HeLa	mice		[211]
Inhibitors targeting gp41 CHR							
Proteins							
FC-1	9.7 ^a^		10	SupT1, PBMC			[120]
N_CCG_-gp41		NA					[122]
ccN28Fd		27.7 ^a^	8	PBMC			[124]
HR121		16.2	1	GHOST			[256]
5-Helix		3.6 ^a^	4	HOS			[261]
Peptides							
DP107		320	1	TZM-bl			[262]
IZN17		22	1	HOS			[159]
N36		509.8 ^a^	8	MT-2			[263]
Inhibitors targeting gp41 6HB							
Proteins							
P20-A		5700 ^a^	14	MT-2, TZM-bl			[131]
C60		12200 ^a^	2	MT-2, TZM-bl			[133]
Inhibitors targeting gp41 MPER							
Proteins							
2F5	14.6 ^c^		177	TZM-bl	NHP/mice	Ⅱ	[138,237]
4E10	1.93 ^c^		181	TZM-bl	NHP	Ⅱ	[138]
10E8	0.35 ^c^		180	TZM-bl	NHP	I	[138,236]
10E8v2.0/iMab	0.002 ^c^		118	TZM-bl	mice		[140]
10E8.4/iMab	0.0008 ^c^		118	TZM-bl	mice	I	[141]
Inhibitors targeting both gp120 and gp41							
8ANC195	NA				mice		[264]
35O22	0.056 ^b^		181	TZM-bl			[220]
VRC34.01	0.35 ^b^		16	TZM-bl			[221]
PGT151	0.008 ^c^		77	TZM-bl			[222]
ACS202	0.14 ^c^		75	TZM-bl			[224]
3BC176	1.69 ^c^		25	TZM-bl			[226]
Griff37		0.08 ^a^	4	PBMC			[234]
C37CD4M33_C1F23_		2.29 ^a^	2	TZM-bl			[234]
2DLT		14 ^a^	5	MT-2, TZM-bl, PBMC			[229]
sCD4-FIT45	0.12 ^c^		16	TZM-bl			[233]

^a^ Arithmetic mean; ^b^ Geometric mean; ^c^ median IC_50_; ^d^ Geometric mean IC_80;_ NA, not applicable; Non-human primate (NHP).
